# A minimally invasive immunocytochemical approach to early detection of oral squamous cell carcinoma and dysplasia

**DOI:** 10.1038/sj.bjc.6603066

**Published:** 2006-04-18

**Authors:** I S Scott, E Odell, P Chatrath, L S Morris, R J Davies, S L Vowler, R A Laskey, N Coleman

**Affiliations:** 1 MRC Cancer Cell Unit, Hutchison/MRC Research Centre, Hills Road, Cambridge CB2 2XZ, UK; 2 Department of Oral Pathology, King's College London Dental Institute, Floor 28, Guy's Hospital Tower, Guy's Hospital, London SE1 9RT, UK; 3 Department of Ear Nose and Throat Surgery, Royal National Throat, Nose & Ear Hospital Grey's Inn Road, London WC1X 8DA, UK; 4 Centre for Applied Medical Statistics, Department of Public Health and Primary Care, University Forvie Site, Robinson Way, Cambridge CB2 2SR, UK

**Keywords:** oral cavity, squamous cell carcinoma, dysplasia, screening, cytology, minichromosome maintenance proteins

## Abstract

Squamous dysplasia of the oral cavity indicates increased risk of progression to squamous cell carcinoma (SCC). An important advance would be the development of a minimally invasive assay for identification of oral SCC and dysplasia. We have investigated the suitability in this context of immunostaining oral smears for minichromosome maintainance proteins (MCMs), sensitive and specific biomarkers of cell cycle entry. Immunohistochemical examination of 66 oral tissue samples showed a greater frequency of Mcm-2 expression in surface layers of moderate/severe dysplasia and SCC compared to benign keratosis/mild dysplasia. Immunocytochemistry for Mcm-2/Mcm-5 was performed on 101 oral smears. Conventional smears included 23 from normal mucosa, benign proliferative disease and mild dysplasia, all of which were MCM negative. Of 52 conventional smears of SCC tissue samples, 18 were inadequate. However, MCM-positive cells were present in 33/34 adequate samples. Of 26 liquid-based cytology smears, 19 out of 20 smears from SCC were adequate and all were MCM positive. Six smears from benign lesions were adequate and MCM negative. We conclude that MCMs are promising markers for early detection of oral SCC and dysplasia, particularly in a liquid-based cytology platform. Detection of MCMs would be amenable to automation and potentially applicable in the developing world. Further studies are now warranted.

Oral squamous cell carcinoma (SCC) is the sixth most common malignancy worldwide. Up to 500 000 new cases of oral and pharyngeal cancer are diagnosed annually and approximately 75% of these occur in the developing world (Moore *et al*, 2000). The most common precursor lesions are seen as white patches of the oral mucosa, although only a minority of these will progress to SCC. While frank SCCs can usually be identified readily on oral examination, clinical differentiation of premalignant lesions and early SCCs from benign proliferative conditions is difficult, in view of the paucity of specific identifying features (Vokes *et al*, 1993).

It has been argued gross genomic aberrations may identify lesions destined to progress to SCC (Lippman *et al*, 2005). However, detecting such abnormalities requires specialist techniques, for example image cytometry, that may not be universally applicable. As an alternative, it has long been recognised that the presence of dysplasia is associated with increased risk of progression to SCC. Detection of dysplasia by biopsy and histopathology requires trained surgeons and pathologists, leading to underinvestigation in developing countries and overinvestigation and associated morbidity in developed countries. Moreover, histological assessment of biopsies is subjective and prone to inter- and intraobserver variation. An important advance would be the development of a minimally invasive, objective test for the identification of oral malignancy and dysplasia, based on cytological sampling of the oral mucosa. Mucosal smears could be performed by nonspecialists without significant morbidity and would enable cost-effective testing that could conceivably be applied to screening at risk populations.

Conventional cytological examination of exfoliated cells may enable diagnosis of some cases of oral SCC but such techniques have a high false positive rate (Dabelsteen *et al*, 1971). At present there is considerable interest in exploiting improved understanding of the biology of cancer cells to develop biomarkers capable of distinguishing malignant and dysplastic cells from their normal counterparts. Importantly, any screening test based on biomarkers could be amenable to automation, producing substantial cost savings and the potential for applications in the developing world. In this regard, we are investigating the clinical value of antibodies against minichromosome maintenance (MCM) proteins in improving the detection of neoplastic cells in cytological samples.

Minichromosome maintenance proteins 2–7 are essential for eucaryotic DNA replication (Kearsey and Labib, 1998; Gonzalez *et al*, 2005). All six proteins are abundant throughout the cell cycle but are broken down rapidly on differentiation and more slowly in quiescence (Kearsey *et al*, 1996; Maiorano *et al*, 1996; Musahl *et al*, 1998; Tanaka and Diffley, 2002; Gonzalez *et al*, 2005), making them sensitive and specific markers of cells that have entered the cell cycle. Antibodies against MCMs detect more cells in tissues than other ‘proliferation’ markers such as Ki67 and proliferating cell nuclear antigen (Freeman *et al*, 1999; Chatrath *et al*, 2003; Scott *et al*, 2003). There is deregulated expression of MCMs 2–7 in malignant/dysplastic epithelia, including in the surface cells sampled in exfoliative cytology (Williams *et al*, 1998; Freeman *et al*, 1999; Chatrath *et al*, 2003). We have previously exploited these findings to develop tests for improved identification of neoplastic cells from a number of anatomical sites, including cervix, colon and larynx (Williams *et al*, 1998; Davies *et al*, 2002; Chatrath *et al*, 2003).

In the present study, we have examined the potential utility of MCMs as candidate biomarkers for detecting oral malignancy and dysplasia in smears of oral lesions. We initially performed immunohistochemical examination of 66 samples of oral mucosal tissue, in which we observed striking differences in expression of Mcm-2 in the surface layers of epithelium showing severe/moderate dysplasia or SCC, compared to mildly dysplastic or benign lesions. Based on this data we undertook MCM immunocytochemistry of 101 oral smears. Taken together, our findings suggest that immunocytochemical detection of MCMs can form the basis of an effective minimally invasive screening test for oral malignancy and dysplasia.

## MATERIALS AND METHODS

### Tissue samples

Blocks of paraffin-embedded, formalin-fixed oral biopsies were obtained in accordance with Local Research Ethics Committee approval. Histopathological diagnosis was made by consensus between two and in many cases three histopathologists, using criteria described in detail elsewhere (Odell and Morgan, 1998). The tissues examined represented benign keratosis (*n*=9); mild (*n*=17), moderate (*n*=16) and severe (*n*=14) dysplasia; and SCC (*n*=10).

### Immunohistochemistry

Sections (5 *μ*m) were cut onto aminopropyltriethoxysilane (APES)-coated slides and processed for immunohistochemistry as described previously (Freeman *et al*, 1999; Chatrath *et al*, 2003; Scott *et al*, 2003). We used mouse monoclonal primary antibodies against Mcm-2 (Mukherjee *et al*, 2001; Chatrath *et al*, 2003), Mcm-5 (Mukherjee *et al*, 2001) and Ki67 (Mib-1 clone, DAKO, Ely, UK). Briefly, primary antibody (100 *μ*l) was applied in a humidified chamber at 4°C overnight with gentle shaking in 1% BSA/TBS with 0.1% Triton X-100. The slides were then washed in TBS containing 0.025% Triton X-100 and incubated for 1 h with biotinylated goat anti-mouse secondary antibody (DAKO, Ely, UK). A streptavidin-horseradish peroxidase system (DAKO, Ely, UK) with the substrate diaminobenzidine was used to develop the stain. The slides were then lightly counterstained with Harris' haematoxylin, dehydrated in increasing concentrations of alcohol and cleared in xylene. Coverslips were applied with DEPEX mounting medium (Gurr, BDH, Poole, Dorset, UK). Negative controls were performed for all tissues by omitting the primary antibody. Sections of cervix showing various grades of intraepithelial neoplasia were used as positive controls (Freeman *et al*, 1999).

### Quantification of antibody staining

For each marker, an indication of staining frequency was determined by calculating a labelling index (LI), representing the ratio of immunopositive to total epithelial cells assessed, counting a minimum of 500 cells per case. Labelling indexes were determined for the entire epithelium and, where epithelial architecture was retained, for four epithelial compartments; the superficial, middle and lower thirds of the epithelium and the basal layer. For each section, the epithelial thirds were defined by measuring the epithelial thickness and dividing by three. In the SCCs, epithelial orientation is lost and we observed uniform expression of the markers studied. A single LI was therefore determined as a mean of five individual counts.

All counts were repeated by three observers (ISS, PC, RJD), and an interobserver variation of less than 5% was seen. The final LI values represented a mean of the LIs from the three observers. We were particularly interested in the LI values for the superficial epithelial third, as the cells in this layer are the ones most likely to be sampled in oral smears.

### Immunocytochemistry of oral smears

Oral smears were obtained from patients attending outpatient clinics at Guy's Hospital, London, with the approval of the Local Research Ethics Committee. Smears were obtained using a blunt rounded metal spatula. We examined 101 oral smears, 75 of which were applied directly to glass slides in the conventional manner and 26 of which were prepared as monolayers by liquid-based cytology (LBC). The findings from subsequent histological evaluation of the sampled epithelium were available in all cases.

The 75 conventional smears were prepared and stained as described previously (Williams *et al*, 1998). While the majority of samples were stained using a monoclonal antibody against Mcm-2 (65 cases), we also included 10 cases stained with a polyclonal antibody against Mcm-5, as the distribution of Mcm-2 and Mcm-5 is essentially identical in tissues from a large number of anatomical sites (Freeman *et al*, 1999). The 75 conventional smears included 23 from tissue shown by biopsy to represent normal mucosa (*n*=3); benign proliferative disease including lichen planus, keratosis, pemphigoid and hairy leukoplakia (*n*=17); and mild dysplasia (*n*=3). The remaining 52 conventional smears were from fresh resection specimens for SCC.

For the 26 cases examined by LBC, the spatula was rinsed in PreserveCyt solution (Cytyc Corporation, Boxborough, MA, USA). The solution was then filtered through a TransCyt filter and the cellular material deposited on a slide in a ThinPrep 2000 cytoprocessor according to the manufacturer's protocols (Cytyc Corporation, Boxborough, MA, USA) (Chatrath *et al*, 2003). Immunocytochemical staining of the slides was performed on a DAKO Universal Stainer (DAKO, Ely, Cambs, UK). The slides were treated with 4 mM sodium deoxycholate and washed in TBS. An equal mixture of primary antibodies against Mcm-2 and Mcm-5 was used and the DAKO Chem Mate HRP-detection kit was employed for all subsequent steps. The slides were counterstained with a modified Papanicolaou method (Williams *et al*, 1998) on a Leica Autostainer XL (Leica, Houston, TX, USA) and were then mounted in DPX.

All test slides were scored by three observers (ISS, LSM, PC), who were unaware of the clinico-pathological diagnosis and of each other's findings. A slide was considered adequate if epithelial cells were present and was scored as positive if one or more MCM-expressing epithelial cell was seen. There was no discrepancy in the opinions of the three observers. Results were compared with the histological diagnosis for the accompanying tissue samples.

### Statistical analysis

Differences between Mcm-2 and Ki67 LIs were compared using the Bland Altman limits of agreement analysis. Labelling index values were compared using the Friedman test, and pairwise comparisons were made using the Wilcoxon Signed Rank test. Differences in LIs in the progression from normal oral mucosa through the various grades of dysplasia to SCC were assessed using the Jonckheere–Terpstra test. Analysis was carried out using SPSSv11 (SPSS inc, Chicago).

## RESULTS

### Tissue sections

**Figure 1:**
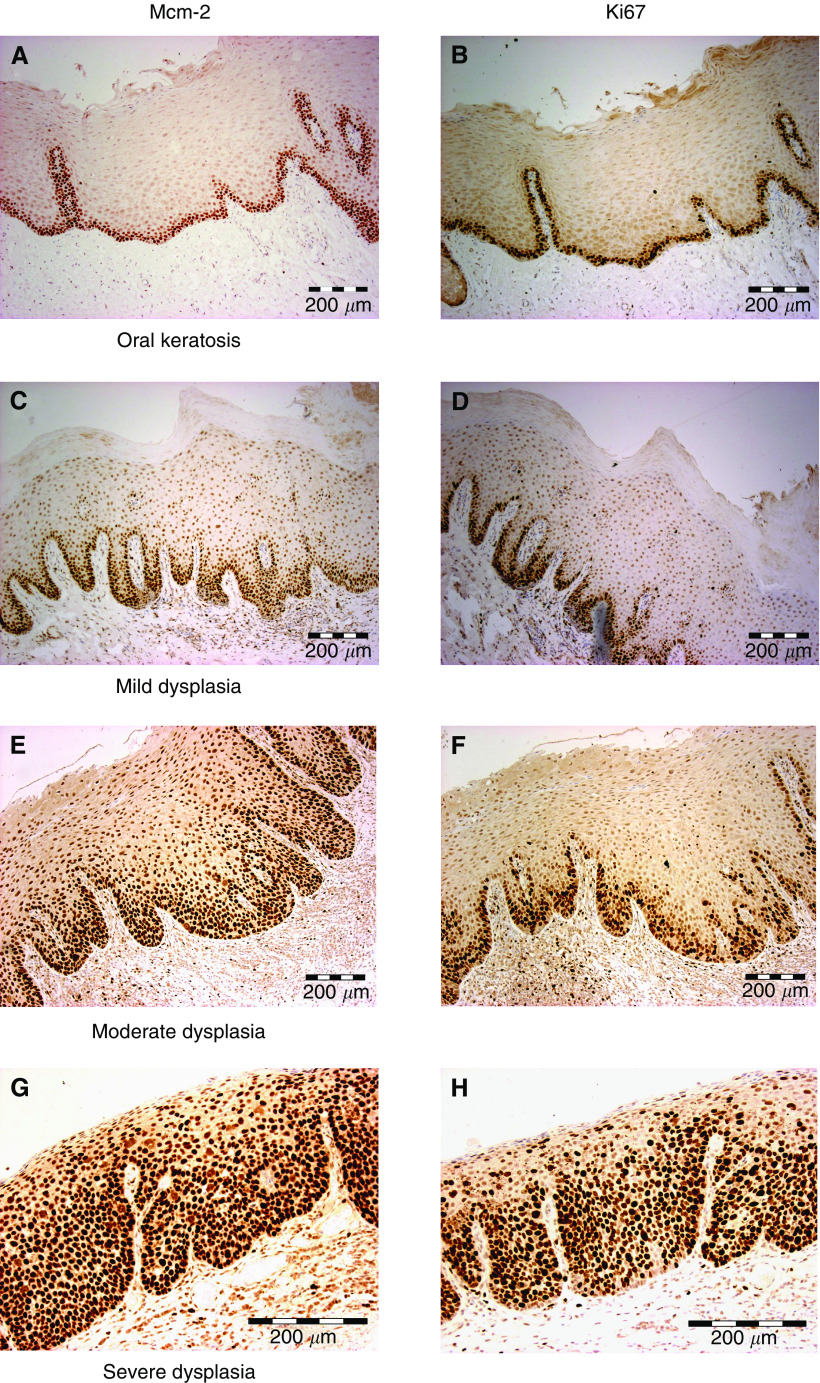
Distribution of Mcm-2 and Ki67 in oral lesions. Immunohistochemical staining illustrating the distribution of Mcm-2 (left) and Ki67 (right) in biopsies of oral mucosa showing benign keratosis and mild, moderate and severe dysplasia. All images are at × 200 magnification.

**Figure 2:**
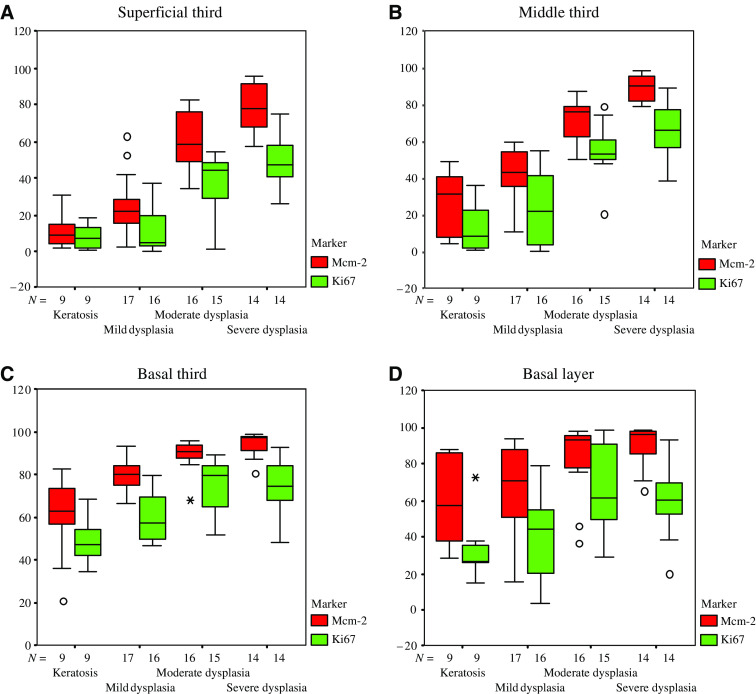
Frequency of Mcm-2 and Ki67 expression in oral lesions. Box plots showing labelling indices for Mcm-2 and Ki67 in different compartments of the oral epithelium in benign keratosis and in mild, moderate and severe dysplasia. Bars=median; boxes=IQR; whiskers=range of data or 1.5 IQRs from the end of the box; circles=outliers; stars=extreme outliers.

In benign keratosis Mcm-2 and Ki67 were generally restricted to basal proliferative compartments (Figure 1), similar to findings in stratified squamous epithelia at other anatomical sites (Freeman *et al*, 1999; Chatrath *et al*, 2003). In regions showing surface ortho- and para-keratosis, expression of both markers also extended to more superficial cells (Figure 2).

**Figure 3:**
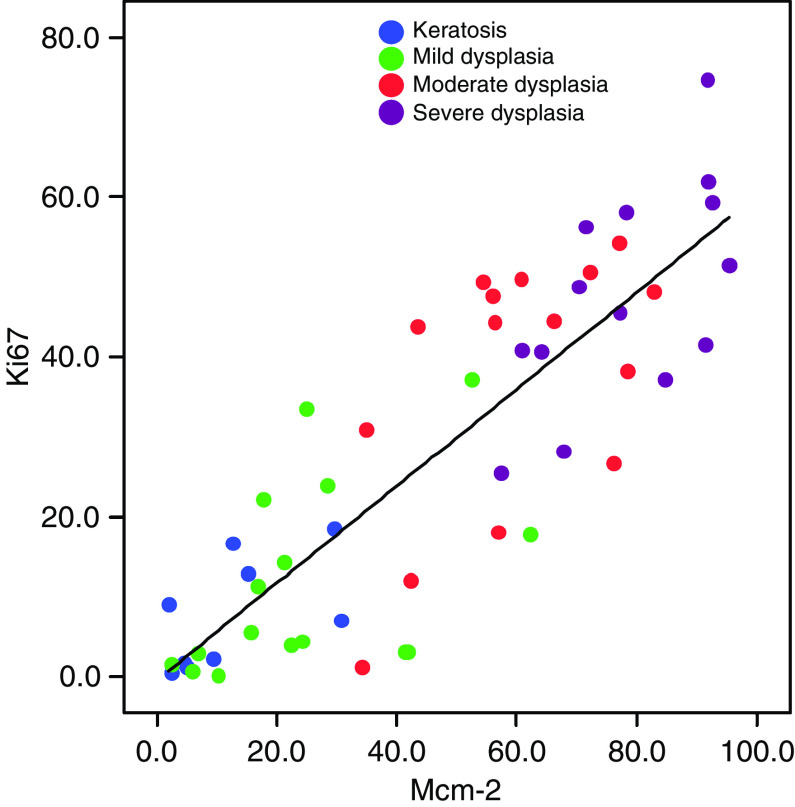
Comparison of Mcm-2 and Ki67 labelling indices in the superficial epithelial third of oral lesions. There is a positive correlation between Mcm-2 and Ki67 LI values for the superficial third of the epithelium in benign keratosis (*n*=9), and in mild (*n*=17), moderate (*n*=16) and severe (*n*=17) dysplasia (*ρ*=0.85; *P*<0.0001).

In dysplasia Mcm-2 and Ki67 were expressed at a higher frequency in all layers of the epithelium (Figure 1). The Mcm-2 LIs and Ki67 LIs showed similar patterns of variation between samples, although the overall Mcm-2 LI values were consistently higher than those for Ki67 (Figure 2). In the stages from keratosis through mild, then moderate to severe dysplasia, Mcm-2 was expressed by increasing numbers of cells in all four epithelial compartments analysed (Figure 2), but the most striking increase was in the superficial layers (*P*<0.0001, Jonkheere–Terpstra test), the source of cells most likely to be sampled in an oral smear. The Mcm-2 LI for this layer (and, to a lesser extent, the Ki67 LI) showed a distinct division between lesions showing benign keratosis or mild dysplasia and those showing moderate or severe dysplasia (Figure 2). Median Mcm-2 LI values were 9.2% for benign keratosis and 21.6% for mild dysplasia, compared with 58.8% for moderate dysplasia and 77.7% for severe dysplasia. There was a positive correlation between Mcm-2 LI and Ki67 LI values in the superficial epithelial third for all samples combined (Spearman's *ρ*=0.85, *P*<0.0001) (Figure 3), with the Mcm-2 LI being significantly greater than the Ki67 LI in the superficial epithelial third in severe (*P*=0.001), moderate (*P*=0.001) and mild dysplasia (*P*=0.004, Wilcoxon signed rank test) (Figure 2).

In SCC there was very widespread expression of Mcm-2 and Ki67, with an overall Mcm-2 LI of 92% (range 80–98%). In keeping with our findings at other anatomical sites (Freeman *et al*, 1999), the highest LI values were observed in the least differentiated SCCs. Similarly high Mcm-2 LI values were seen in the surface layers in all cases. Moreover, small clumps of sloughed immunopositive epithelial cells were frequently identified at the surface of SCCs, reminiscent of our findings in malignancies at other sites (Davies *et al*, 2002).

### Oral smears

In view of the high frequency of Mcm-2 expression in the superficial third of moderate and severe dysplasia and in the surface layers of SCCs, a pilot study was undertaken to investigate whether SCCs could be distinguished from mild dysplasia and benign keratosis by immunocytochemical analysis of MCM proteins in 101 smears of oral mucosa.

**Figure 4:**
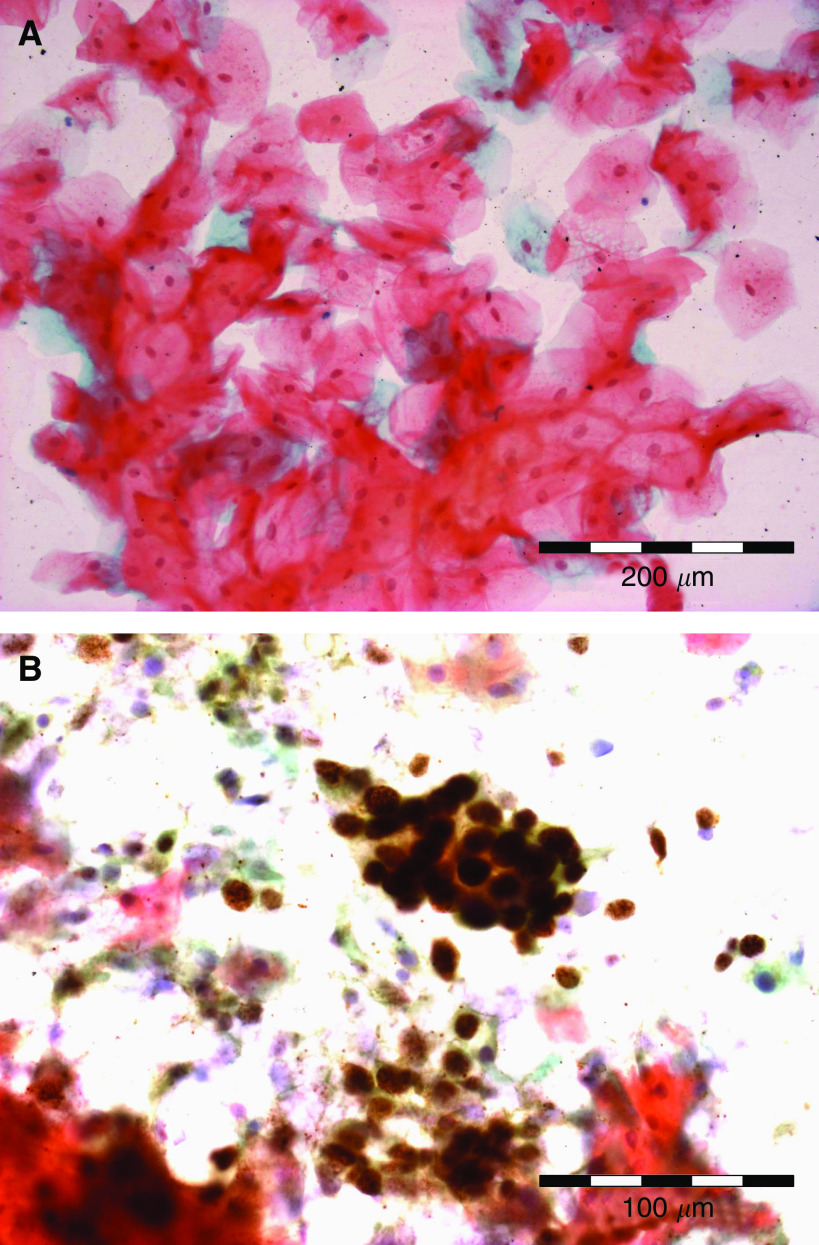
MCM immunocytochemistry in oral smears. Squamous epithelial cells from normal mucosa (**A**) are negative when stained with antibodies against MCM 2/5. In sharp contrast, a cluster of cells from an oral SCC (**B**) are strongly positive for MCM 2/5 and can be identified readily, even at low magnification.

Of the 75 conventional smears examined, all samples taken from the 23 cases of normal mucosa, benign proliferative disease and mild dysplasia contained epithelial cells and were MCM negative (Figure 4A). Of the 52 conventional smears taken from cases of SCC, 18 proved inadequate for assessment, as they did not contain epithelial cells. Of the 34 adequate smears, 33 contained MCM-positive cells, which were readily identified, even at low magnification (Figure 4B). In many cases, the counterstain enabled cytological features of malignancy to be confirmed in the immunopositive cells. Morphologically normal cells were MCM negative. The one MCM-negative adequate smear from a SCC represented a case of recurrent malignancy following radiotherapy, where the biopsy showed stromal scarring and no malignant cells appeared to have been sampled in the smear.

Of the 26 smears examined by LBC, 20 were from SCC. Only one of these cases proved inadequate for assessment. MCM-positive cells were present in all 19 of the adequate samples. Moreover, all six LBC preparations from patients with benign lesions were adequate and MCM negative. The LBC samples also produced greater consistency of staining and ease of interpretation compared to the conventional smears.

## DISCUSSION

Numerous features of oral cancer make it well suited to early detection through screening, subject to the availability of a suitable and reliable test. Oral cancer is one of the most common malignant lesions of the head and neck, particularly in developing countries, where large populations are exposed to irritant surface carcinogens such as tobacco smoke and betel nut extracts (Weiss and Goldblum, 2001). Generally, there is progression through increasing grades of epithelial dysplasia to invasive malignancy. Although tumours of the oral cavity usually present with relatively low-volume disease compared with those of the oropharynx, cancers of the buccal lining, the most common subgroup affecting Asian and African communities, typically present at a late stage owing to the relative insensitivity of this part of the mouth (Watkinson *et al*, 2000). In addition, treatment protocols involving surgery and/or chemotherapy are disfiguring and associated with considerable morbidity.

The nonspecific clinical appearances of dysplastic and early malignant lesions in the oral cavity further emphasise the need to develop effective methods for earlier detection of such lesions. Unlike the larynx, where a similar pattern of neoplastic progression is observed and where the need for minimally invasive approaches is arguably just as pressing (Chatrath *et al*, 2003), the oral cavity is more easily accessible for tissue sampling, tumour surveillance and post-treatment monitoring, without a requirement for expensive flexible nasoendoscopic equipment or advanced expertise. Indeed, screening of the oral cavity using LBC could conceivably be performed using a self-administered mouthwash technique.

One of the features of epithelial dysplasia and malignancy is ectopic cell cycle entry. As key proteins of the prereplication complex, MCMs are sensitive and specific markers of cells in cycle. Indeed, we have previously suggested that deregulated expression of these proteins may characterise the dysplastic and malignant states (Freeman *et al*, 1999). In studies of neoplastic lesions at other anatomical sites, MCMs have consistently been more abundant than other putative markers of cell cycle entry, such as Ki67 (Gonzalez *et al*, 2005). Our present data is consistent with such observations and suggests that MCMs are likely to be more sensitive biomarkers for cytological diagnosis of oral malignancy and dysplasia than Ki67.

Our cytological data are fully consistent with our histopathological observations and indicate that MCM-positive epithelial cells are likely to be present in smears from oral SCC but not in scrapes of mild dysplasia and benign keratosis. In the present study, we did not test directly the value of MCM immunocytochemistry in detecting severe/moderate oral dysplasia. However, the high frequency of expression of Mcm-2 in surface layers in histological sections of these conditions suggests that the detection sensitivity in smears is likely to be very high. Taken together, our data suggests that the value of MCM immunocytochemistry in the analysis of oral smears is likely to be similar to that for other cytological samples, such as smears of the cervix and larynx, where detection of MCMs enables dysplastic and malignant cells to be detected with a high degree of sensitivity and specificity (Williams *et al*, 1998; Chatrath *et al*, 2003).

We have not investigated whether MCMs enable identification of the approximately 20% of dysplastic oral lesions that progress to SCC. The presence of gross genomic abnormalities, manifesting as tetraploidy or aneuploidy, has been claimed to be a useful indicator of lesions destined to progress (Lippman *et al*, 2005). It is likely that epithelia showing cell cycle dysregulation also show genomic instability, leading to aberrations in DNA content. Cell cycle markers may therefore be useful progression markers, either independently or as surrogate indicators of gross genomic imbalances. Indeed, it may be easier for routine diagnostic laboratories to measure cell cycle markers than DNA indices. It will be worth investigating the relationships between aneuploidy in oral lesions and cell cycle dysregulation, as demonstrated by ectopic expression of cell cycle markers. As well as indicators of cell cycle ‘state’, such as MCMs, the latter should also include markers of cell cycle progression such as geminin, which is present only in the later stages of the cell cycle and provides an indication of cell cycle ‘rate’ in clinical samples (Gonzalez *et al*, 2005).

While our findings using conventional oral smears are broadly comparable to those obtained with LBC, the latter appears to reduce substantially the frequency of inadequate samples. As well as providing overall cost benefits, this would be an important practical advantage in some settings, including the developing world, where it may be difficult for individuals to reattend for repeat investigations should an initial sample prove inadequate. The strong nuclear signal provided by MCMs makes identification of immunopositive cells straightforward and should facilitate the development of approaches to automated analysis of immunostained slides.

In summary, MCM immunocytochemistry holds great promise as a minimally invasive test for early detection of oral malignancy and dysplasia. The strong clinical performance and ready interpretation of stained LBC samples makes MCMs particularly strong markers for high-throughput screening of high-risk patients. Indeed, the method may be applicable to the developing world where the incidence of oro-pharyngeal SCC is high (Vokes *et al*, 1993). In this regard, oral washings may yield adequate numbers of cells from many early lesions and thereby permit self-sampling. Larger scale studies of this promising approach, including assessment of correlations with potential markers of progression such as aneuploidy, are now warranted.
